# 

**Published:** 1905-02

**Authors:** 


### BOOKS RECEIVED.

Annual Report of the Surgeon-General of the Public Health and Marine Hospital Service of the United States. For the fiscal year 1904. Walter Wyman, M.D., Surgeon-General. Washington: Government Printing Office.

Transactions of the Twenty-sixth annual meeting of the American Laryngological Association, held at Atlantic City, N. J., June 2, 3 and 4, 1904. James E. Newcomb, M.D., Secretary. New York: Rooney & Otten Printing Co.

A Compend of the Practice of Medicine. By Daniel E. Hughes, M.D., formerly Demonstrator of Clinical Medicine in the Jefferson Medical College of Philadelphia. Seventh revised edition by Samuel Horton Brown, M.D., Assistant Dermatologist, Philadelphia Hospital. Including sections on Mental and Skin Diseases. Duodecimo, pp. 779. Illustrated. Philadelphia: P. Blakiston's Son & Co. 1904. (Price, flexible leather, $2.50.)

Blakiston's Quiz Compends. A Compend of the Diseases of the Eye and Refraction. By George M. Gould, Editor of American Medicine and Walter L. Pyle, Assistant Surgeon to Wills Eye Hospital, Philadelphia. Third edition, revised and corrected. One hundred and nine illustrations, some in colors. Philadelphia: P. Blakiston's Son & Co. 1904. (Price, $1.00.)

The Diseases of Society (The Vice and Crime Problem). By G. Frank Lydston, M.D., Professor of Genitourinary Surgery, State University of Illinois. Small octavo, pp. 626. Illustrated. Philadelphia and London: J. B. Lippincott Co. 1904. (Price, $3.00.)

Pneumonia and Pneumococcus Infections. By Robert B. Preble, M.D., Professor of Medicine, Northwestern University. Duodecimo, pp. 211. Illustrated. Chicago: Cloyd J. Head & Co. 1905. (Price, $1.00.)

Transactions of the American Surgical Association. Vol. XXII. 1904. Edited by Richard H. Harte, M.D., Recorder of the Association. Philadelphia: Wm. J. Dornan, Printer. 1904.

The Surgical Diseases of the Genitourinary Tract, Venereal and Sexual Diseases. A Textbook for Students and Practitioners. By G. Frank Lydston, M.D., Professor of the Surgical Diseases of the Genitourinary Organs and Syphilology in the Medical Department of the State University of Illinois (the College of Physicians and Surgeons). Second revised edition. Illustrated with 233 engravings and 7 colored plates. Pages xv.-1008. Philadelphia: F. A. Davis Company. 1904. (Cloth, $5.00 net; sheep or half-russia, $6.00 net.)

A Treatise on Diseases of the Nervous System. By L. Harrison Mettler, M.D., Associate Professor of Neurology, College of Medicine of the University of Illinois. Octavo, 1,000 pages. Illustrated, including two color plates. Chicago: Cleveland Press. 1904. (Cloth, $5.00; half morocco, $6.00.)

Textbook of Insanity based on Clinical Observations. For Practitioners and Students of Medicine. By Dr. R. von Krafft-Ebing, late Professor of Psychiatry and Nervous Diseases in the University of Vienna. Authorized Translation from the Latest German Edition by Charles Gilbert Chaddock, M.D., Professor of Diseases of the Nervous System in the Marion-Sims-Beaumont College of Medicine, Medical Department of Saint Louis University, Saint Louis. With an Introduction by Frederick Peterson, M.D., President of the New York State Commission in Lunacy. Pages xvi-638. Royal octavo. Philadelphia: F. A. Davis Company. 1905. (Cloth, $4.00 net; half russia, $5.00 net.)

